# Chromosome Evolution in Marsupials

**DOI:** 10.3390/genes9020072

**Published:** 2018-02-06

**Authors:** Janine E. Deakin

**Affiliations:** Institute for Applied Ecology, University of Canberra, Canberra, ACT 2617, Australia; janine.deakin@canberra.edu.au; Tel.: +61-2-6206-8663

**Keywords:** cytogenetics, epigenomics, genome evolution, genomics, speciation, wallaby

## Abstract

Marsupials typically possess very large, distinctive chromosomes that make them excellent subjects for cytogenetic analysis, and the high level of conservation makes it relatively easy to track chromosome evolution. There are two speciose marsupial families with contrasting rates of karyotypic evolution that could provide insight into the mechanisms driving genome reshuffling and speciation. The family Dasyuridae displays exceptional karyotype conservation with all karyotyped species possessing a 2*n* = 14 karyotype similar to that predicted for the ancestral marsupial. In contrast, the family Macropodidae has experienced a higher rate of genomic rearrangement and one genus of macropods, the rock-wallabies (*Petrogale*), has experienced extensive reshuffling. For at least some recently diverged *Petrogale* species, there is still gene flow despite hybrid fertility issues, making this species group an exceptional model for studying speciation. This review highlights the unique chromosome features of marsupial chromosomes, particularly for these two contrasting families, and the value that a combined cytogenetics, genomics, and epigenomics approach will have for testing models of genome evolution and speciation.

## 1. Introduction

Two observations led to the idea that chromosome rearrangements may play a role in speciation; chromosome numbers can vary greatly, even between closely related species, and hybrids resulting from crosses of individuals with different chromosome arrangements often experience reduced fertility [1,2]. Whether chromosome rearrangements actively contribute to the speciation process or are an incidental consequence remains unclear and has been much debated in the literature (reviewed in [3]). How chromosome rearrangements occur in the first place is fundamental to understanding genome evolution. Do rearrangements occur randomly across the genome or are certain regions prone to rearrangement and if so do these regions have common features? These are the questions that have driven genome evolution research in recent years (reviewed in [4]). Comparisons of different sequenced vertebrate genomes have supported a “fragile breakage model” for genome evolution, identifying regions of the genome prone to breakage [5,6,7]. In mammalian genomes, these fragile regions are enriched for repetitive sequences, including segmental duplications, are often gene dense, and are actively transcribed (reviewed in [4]). Recently, the “integrative breakage model” was proposed. This model not only considers the sequence content but also takes into account gene function, epigenomic features, and chromatin conformation of regions involved in chromosome rearrangements [4]. The model posits that regions prone to genomic reshuffling have an open chromatin conformation, are enriched with actively transcribed genes but not those with an essential function, and are regions in physical contact within the nucleus [4]. This model is supported by fine-scale comparisons of rodent genomes with those of outgroup species where evolutionary breakpoints were gene-rich and had an epigenetic profile of open, actively-transcribed chromatin [8].

The challenge is finding appropriate groups of study species on which to test models of genome evolution and speciation. One group of mammals that may be ideal for these purposes is the infraclass Metatheria—the marsupials. Here, I highlight the unique features of marsupial chromosomes and how these features could help to address questions regarding the mechanisms involved in genome reshuffling and the role that rearrangements play in speciation. 

## 2. Marsupial Chromosome Evolution

**Figure 1:**
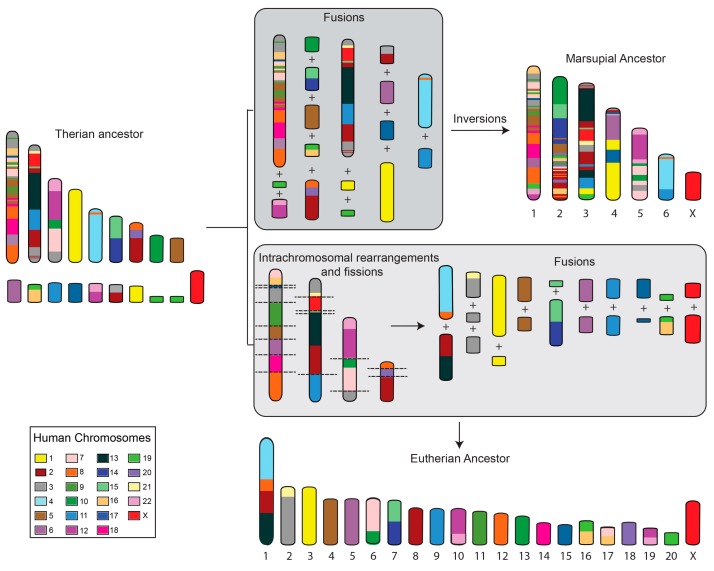
Predicted events leading to the proposed ancestral marsupial and eutherian karyotypes. Chromosomes have been color-coded to reflect their homology to human chromosomes. Chromosome fusions followed by inversions resulted in a 2*n* = 14 marsupial ancestral karyotype [14] whereas a series of intrachromosomal rearrangements followed by fissions and fusions resulted in the predicted 2*n* = 42 ancestral eutherian karyotypes [18].

There are over 300 species of marsupials that are split between the Americas with approximately 100 species (Ameridelphia) and Australasia, consisting of just over 200 species (Australidelphia). The Ameridelphia and Australidelphia last shared a common ancestor around 80 million years ago [9]. Marsupials, with their large chromosomes and small diploid numbers, were among the first mammals to have their chromosomes observed in the early part of last century and their chromosomes were noted to be particularly amenable to being studied [10,11,12,13]. The ancestral marsupial is proposed to have possessed a 2*n* = 14 karyotype, similar to the karyotype observed in many species distributed across the marsupial phylogeny [14,15,16]. The large marsupial chromosomes are predicted to have arisen mainly from the fusion of chromosomes present in the common ancestor of marsupial and eutherian (therian) mammals (Figure 1) [14]. In contrast, a more complex set of rearrangements, including a series of fission and fusion events, are predicted to have led to a higher chromosome number in the ancestral eutherian (Figure 1). Chromosome complements observed amongst marsupials are easily derived from the predicted marsupial ancestral karyotype by various combinations of fissions, fusions, and centromere repositioning [17]. 

**Figure 2:**
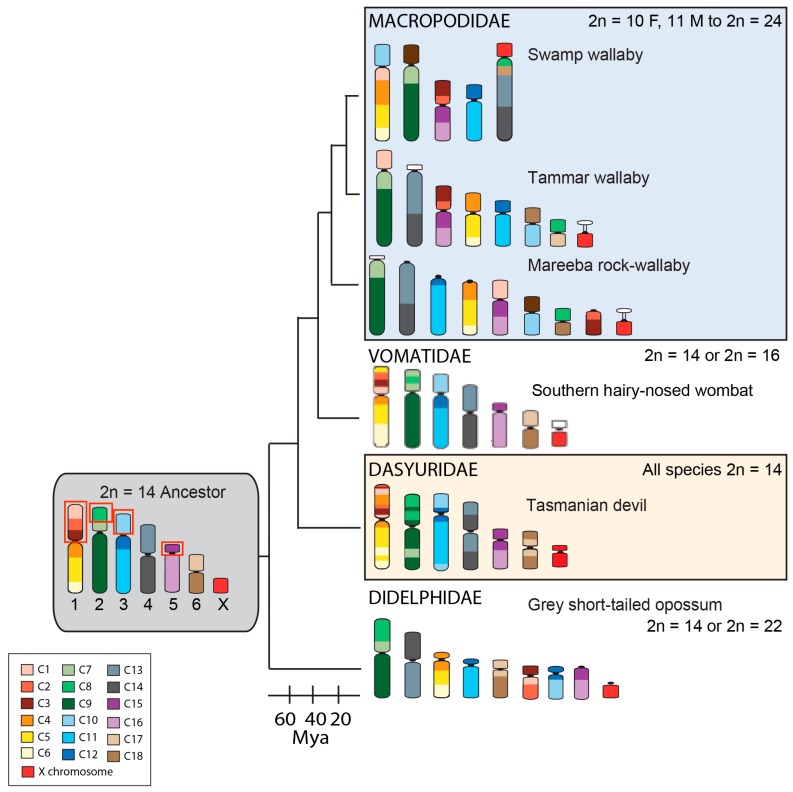
Arrangement of the conserved segments (C1–C19) identified in the ancestral marsupial and in species from different marsupial families. The diploid number range is indicated for each family. Chromosomes are color-coded to show the arrangement of the 19 conserved segments identified by chromosome painting [20]. The conserved segments most commonly rearranged across the marsupial phylogeny are indicated by red boxes on the ancestral karyotype. Mya—Millions of years ago.

Marsupial chromosomes were intensively studied when cytogenetics was at its peak in the 1970s and 1980s [19]. These studies revealed some interesting observations that make marsupials good models for investigating the mechanisms behind chromosome evolution. For instance, the family Dasyuridae, consisting of over 70 species, has remarkable chromosome conservation with all karyotyped members possessing a 2*n* = 14 karyotype (Figure 2), which appears to differ between some species only by intrachromosomal rearrangements. In contrast, the family Macropodidae (kangaroos and wallabies) has experienced considerable genomic reshuffling amongst its 60 odd species. In comparison to eutherian mammals where chromosome numbers can differ markedly between closely related species (e.g., the Indian muntjac with 2*n* = 6 Female (F), 7 Male (M) and Chinese muntjac 2*n* = 46), the marsupial families provide an opportunity to dissect out the mechanisms involved in chromosome evolution and speciation. 

Chromosome painting of distantly related species has shown that marsupial chromosomes are basically divided into 19 conserved segments; 18 autosomal segments and one corresponding to the X chromosome (Figure 2) [20]. Comparisons of the arrangement of these segments between species using chromosome painting make it clear that some chromosomes or chromosomal segments have been particularly prone to rearrangement during marsupial evolution [20]. More detailed comparisons afforded by genome assemblies and dense cytogenetic maps have made it even more evident the extent to which some regions of marsupial chromosomes are prone to breakage. For instance, comparisons of the grey short-tailed opossum (*Monodelphis domestica*) with the Tasmanian devil (*Sarcophilus harrisii*) and tammar wallaby (*Notamacropus eugenii*) show that segments corresponding to the short arms of the ancestral marsupial chromosomes 1, 2, 3, and 5 are more rearranged than other segments [14,21]. When a broader comparison across marsupials is made, it becomes clear that some of these segments are commonly involved in rearrangements, even between species within the family Dasyuridae with its highly conserved karyotype. 

### 2.1. Recombination in Marsupials

The exchange of genetic material between homologous chromosomes is important for the proper segregation of chromosomes during meiosis. A reduced recombination rate, whether a cause or consequence of chromosome rearrangements, is associated with speciation, so it is important to have a basic understanding of recombination rates and the regions involved in chiasmata in marsupials. Cytological studies of marsupial chromosomes during meiosis have reported lower frequencies of chiasmata in females than males. In the opossum, fat-tailed dunnart (*Sminthopsis crassicaudata*), and brushtail possum (*Trichosurus vulpecula*), crossovers occurred more often at the ends of chromosomes in females but were more likely to occur interstitially on male chromosomes [22,23,24]. These sex differences have been supported by linkage mapping data for two of these species, the opossum and the dunnart [22,25]. A linkage map generated for the tammar wallaby also shows a reduced level of recombination in females compared to males but it appears to be localized to specific regions of the genome rather than a genome-wide phenomenon [26,27]. The recombination rates reported for the wallaby and opossum are among the lowest rates reported for vertebrates and the lower female recombination rate in the opossum and other species is opposite to that observed for most eutherians mammals or other vertebrates [28]. The brushtailed bettong (*Bettongia penicillata*) is an exception with no difference in chiasma observed between males and females [29]. The reason for the low recombination rate reported for most species examined or why there is a lower female to male recombination rate is unknown. A more thorough investigation of recombination in marsupials, both cytologically and by linkage mapping, is warranted. In addition, determining the epigenetic profile of female compared to male chromosomes during meiosis may shed light on the differences in recombination rates. 

### 2.2. Marsupial Sex Chromosomes

The X chromosomes of marsupials vary substantially in size, morphology, and banding pattern, even between species with an ancestral-like 2*n* = 14 karyotype [16,19]. In most species, chromosome painting has demonstrated that the X consists of a single conserved segment, sharing homology with two-thirds of the human X [20,30]. The remaining third of the human X is autosomal in marsupials, corresponding to part of chromosome 3 in the ancestral marsupial [14,31]. The marsupial X, therefore, represents the ancestral therian X chromosome to which an autosomal chromosome segment was added to after the divergence of the marsupial and eutherian lineages (Figure 1) [32]. Among even the most distantly related eutherians, gene content and gene order are typically highly conserved, with rodents and cetartiodactyls being exceptions [33,34]. In contrast, gene order on the X chromosome of marsupials is not conserved, with a high degree of rearrangement being observed between opossum, devil and tammar wallaby X chromosomes [31,35]. In rodents and cetartiodactyls, rearrangements of the X chromosome have been proposed to be associated with repetitive and/or duplicated sequences, which may have facilitated rearrangement [34,36]. The fragmented nature of the genome assemblies for the devil and tammar wallaby have made an analysis of the repetitive sequences occurring at breakpoints on marsupial X chromosomes difficult at this stage. However, it will be interesting to test this hypothesis for eutherian species having similarly experienced X chromosome rearrangements using marsupial genome data. 

Ohno’s law posited that translocations between the X and autosomes would be selected against to avoid disrupting the dosage compensation mechanism [37]. However, translocations or fusions between autosomes and the sex chromosomes have been observed in a number of eutherian species (reviewed in [38]), as well as in several marsupial species including the swamp wallaby (*Wallabia bicolor;* 2*n* = 10 XX F 2*n* = 11 XY_1_Y_2_ M), the long-nosed potoroo (*Potorous tridactylus*; 2*n* = 12 XX F, 2*n* = 13 XY_1_Y_2_ M), greater bilby (*Macrotis lagotis*), and spectacled hare-wallaby (*Lagorchestes conspicillatus;* 2*n* = 18 F X_1_X_1_X_2_X_2_, 2*n* = X_1_X_2_Y M*)* (reviewed in [18]). Chromosome painting has verified the chromosomes involved in sex chromosome-autosome fusions for the swamp wallaby [39] and potoroo [20]. The short arm of the swamp wallaby X chromosomes is homologous to the X of other marsupials, whereas the long arm of the X and the entire Y_2_ share homology with ancestral marsupial chromosomes distal 1p, distal 2p, and the entire chromosome 4 [38]. Chromosome painting with the tammar wallaby Y chromosome showed that Y_1_ is homologous to the tammar wallaby Y chromosome but there are also some shared sequences on Y_2_ and the X [38]. The lack of gene order and the finding of several species with X-autosome fusions may be related to the differences in the dosage compensation mechanism between marsupials and eutherians. 

Like eutherians, marsupials inactivate one X chromosome in female somatic cells as part of the dosage compensation mechanism to equalize expression between males with one X and females with two. However, there are some striking differences in the X inactivation mechanism between marsupials and eutherians. Marsupials preferentially inactivate the paternally-derived X and inactivation is often incomplete. This contrasts with the random and more tightly controlled inactivation observed in eutherians (reviewed in [40]). Furthermore, the long non-coding RNA, X-inactive specific transcript (*XIST),* which plays a critical role in X inactivation in eutherians, is absent in marsupials. Instead, marsupials have independently evolved a non-coding RNA called *RSX* (RNA on the silent X) that appears to perform the equivalent role of *XIST* [41]*.* Although there are some shared epigenetic marks between marsupial and eutherian X inactivation, such as the depletion of marks associated with active chromatin from the inactive X (e.g., H3K4me2, H4Kac, H3K9ac) and enrichment of the repressive marks H3K27me3 and H3K9me2 (reviewed in [40]), there are additional epigenetic modifications that may be responsible for the more complete and stable inactivation observed in most eutherians. The more incomplete form of inactivation observed in marsupials may enable rearrangements on the X chromosome to be tolerated more so than on the X chromosome of eutherians. Very few species, either marsupial or eutherian, have had their X inactivation mechanisms studied in detail. Comparisons of marsupials with eutherian species that have experienced either intra- or interchromosomal X chromosome rearrangement could help to determine whether the X inactivation mechanism plays a role in the level of tolerance of rearrangement. 

The Y chromosome in marsupials is typically very small, being no more than 12 Mb in some dasyurids as compared to the ~60 Mb human Y chromosome [42]. The marsupial Y chromosome has been referred to as a “minimal mammalian Y”, carrying the Sex determining region Y gene (*SRY)*, the presumed sex-determining gene in marsupials [42,43]. A set of ten ancestral marsupial Y chromosome genes have been discovered so far by sequencing of bacterial artificial chromosome (BAC) clones mapping to the tammar wallaby and opossum Y chromosomes or identified in a devil testis transcriptome [44,45,46]. In marsupials, the X and Y chromosomes do not share a pseudoautosomal region and, therefore, do not form a synaptonemal complex, nor do the X and Y recombine [47]. In marsupials with sex chromosome-autosome fusions, a synaptonemal complex is formed between the regions homologous to the ancestral marsupial autosomal regions but not between the sex chromosome segments [19]. The absence of X–Y recombination and lack of synaptonemal complex formation may contribute to the high frequency at which the X and Y chromosomes are observed to separate in metaphase I during meiosis [19].

### 2.3. Marsupial Genomics

To date, much of the work on marsupial chromosome evolution has focused purely on a cytogenetic perspective. It is timely that we now revisit this area of research to bring it more fully into the genomics era. Currently, there are five sequenced marsupial genomes: the grey short-tailed opossum (*M. domestica*) [48], Tasmanian devil (*S. harrisii*) [49,50], tammar wallaby (*N. eugenii)* [51], koala (*Phascolarcotos cinereus*) [52], and the extinct Tasmanian tiger (*Thylacinus cynocephalus)* [53]. The opossum, devil, and wallaby genomes have cytogenetic mapping data to anchor sequence to chromosomes [14,21], which is essential if these genomes are to be useful for studying marsupial chromosome evolution. The Oz Mammals Genomics Initiative is currently underway to sequence many more genomes of Australian marsupials distributed across the Australidelphia phylogeny [54], which will provide a much-needed resource for a more detailed study of marsupial chromosome evolution. The stark differences between dasyurid and macropod chromosome evolution make genome assemblies from these families particularly valuable for gaining insight into the drivers of chromosome evolution and for testing the role of chromosome rearrangements in speciation. The sequencing of many more marsupial genomes will make it possible to uncover the features of these chromosome segments that have made them prone to breakage throughout marsupial evolution.

## 3. Dasyurid Chromosome Evolution and Unique Chromosome Features

Dasyurids essentially share the same karyotype with all species studies to date (>40), having almost identical 2*n* = 14 chromosome complements [16,55,56,57,58,59]. This level of karyotypic conservation is remarkable considering their divergence from a common ancestor approximately 55 million years ago. Intrachromosomal rearrangements are the only exceptions to this striking conservation observed by G-banding. These rearrangements are essentially inversions observed in just four species; a paracentric inversion on chromosome 6 of the kultarr (*Antechinomys laniger*), a pericentric inversion on chromosome 6 of the Wongai ningaui (*Ningaui ridei*) and the southern ningaui (*Ningaui yvonneae*) [59], and a pericentric inversion on devil (*S. harrisii*) chromosome 5 [60]. Centromere repositioning has been shown in some mammals to distinguish species (reviewed in [61]). In dasyurids, the only centromere repositioning relates to a couple of pericentric inversions. In *N. yvonneae* and *S. harrisii*, these inversions are polymorphic in the population [59,60] and may, therefore, have little or no effect on fertility of heterozygous individuals. It is important to keep in mind that very few studies have used multiple samples from the same species, so it is unclear whether polymorphisms also exist in populations of other dasyurids. In addition, G-banding is limited in its ability to detect intrachromosomal rearrangements, particularly small-scale rearrangements. For instance, fluorescence in situ hybridization (FISH) of four different BAC clones has detected more chromosome 5 variants amongst devils than the simple pericentric inversion detected by G-banding [62]. Similarly, a comparison of the dense devil cytogenetic map to the chromosome painting data for *Sminthopsis crassicaudata* suggests there has been intrachromosomal rearrangements between these two species [20,21].

Another striking feature of dasyurid chromosomes is a telomere length dimorphism, where one homolog of each chromosome has long telomeres and the other has noticeably shorter telomeres [63]. Such a dimorphism has not been observed in any other group, making dasyurids rather unique. It has been proposed that the paternally-derived haploid set of chromosomes possess long telomeres based on the Y chromosome of five species examined always possessing long telomeres [63]. The maternally-derived chromosomes, therefore, have short telomeres. This parental control of telomere length model suggests resetting of telomere length in the germline, with elongation of telomeres taking place in the male germ line and trimming of telomeres in the female germline [63,64].

It is curious, given the unusual telomeric feature of dasyurid chromosomes that there appears to be an absence of interchromosomal rearrangements in germ cells to generate karyotypic diversity between species. Is there some relationship between the resetting of telomere length in the germline that inhibits interchromosomal rearrangements from occurring? It is tempting to speculate that the more common occurrence of chiasmata at the ends of chromosomes in female *S. crassicaudata*, as opposed to males [22], is related to the difference in their telomere length and the effect this may have on the epigenetic profile of the adjacent region. However, this difference in recombination pattern is not restricted to dasyurids and is observed in more distantly related species such as the opossum and brushtail possum, which do not possess a telomere length dimorphism [23,24,63].

Conversely, this telomere length dimorphism may have played a role in the apparent susceptibility of dasyurids to developing cancers [65,66,67], including two devil facial tumors. Devil facial tumor 1 (DFT1) and devil facial tumor 2 (DFT2) are deadly transmissible tumors decimating the devil population. The erosion of the short telomeres over time may have led to the fusion of the maternal copies of chromosomes 1 and X in DFT1, and chromosomes 1 and 6 in DFT2 [21,62,68]. Why this telomere length dimorphism persists and has not been selected against, given the role it may play in tumorigenesis, is an interesting question.

### Future Directions for Dasyurid Chromosome Research

With their highly conserved karyotypes and unique telomere length dimorphism, dasyurids make a fascinating group of marsupials for gaining insight into genome evolution and speciation. Unfortunately, large comparative studies of their genomes have not been carried out since the original characterization of their chromosomes by G-banding were reported. The dunnart (*S. crassicaudata*) has in the past been used as a model marsupial species because of its ability to be bred in a laboratory setting in a similar fashion to mice and its ability to produce multiple young in one litter [69]. Indeed, the dunnart is gaining traction as a model species with its genome currently being sequenced to hopefully a chromosome-level assembly [54]. This level of assembly will be critical for exploring more fully the level of rearrangement among dasyurids. Although the devil genome has been sequenced and a comparison of these two more distantly related dasyurids would be insightful, a more interesting comparison would be with one of the members of the *Sminthopsis* genus. Have there been chromosome rearrangements between closely related species that are undetectable by the low-resolution G-banding technique? For that matter, are dasyurid chromosomes as highly conserved as proposed based on G-banding? Based on G-banding studies, bird karyotypes, particularly their macrochromosomes, were thought to be highly conserved but a combination of molecular cytogenetic mapping and bioinformatics analysis has demonstrated that these presumably conserved chromosomes have experienced numerous intrachromosomal rearrangements [70,71]. Similar findings are likely should dasyurid genomes be compared using higher-resolution approaches. It would also be useful to carry out more population-based cytogenetic studies on dasyurids to see how widespread chromosome polymorphisms are within and between populations and the effect, if any, these polymorphisms have on fertility.

Microdissection of the tiny dunnart Y chromosome would be ideal for direct sequencing to obtain a Y-specific sequence. Unlike the tammar wallaby Y chromosome, it does not contain repetitive sequences shared with the X or autosomes that would complicate assemblies [42]. It is likely to contain Y-specific repeats but at least there would be one less challenge to overcome for assembly. Obtaining the sequence for another marsupial Y chromosome would provide greater insight into the evolution of this tiny yet important chromosome. 

The telomere length dimorphism raises many questions that may be important for understanding genome evolution in this species. Is there a relationship between the mechanisms involved establishing the dimorphism, presumably in the germline, and the lack of interchromosomal rearrangements between species? The dunnart provides a model to further explore this fascinating phenomenon.

## 4. Macropod Chromosome Evolution

**Figure 3:**
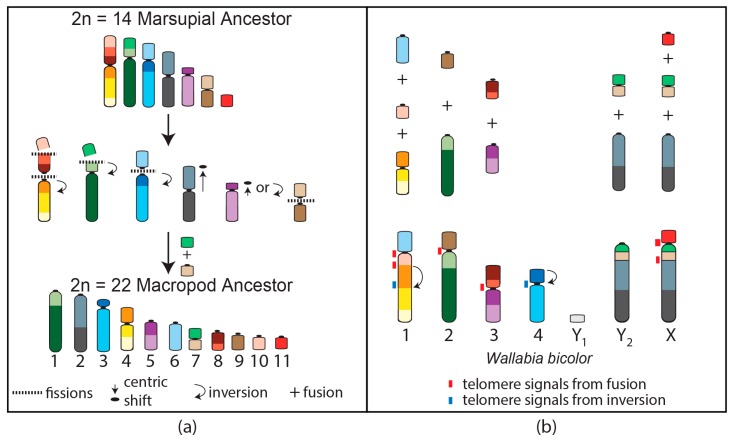
Chromosome rearrangements resulting in the (**a**) macropod 2*n* = 22 ancestral karyotype from the 2*n* = 14 ancestral marsupial and (**b**) the rearrangements of the ancestral macropod chromosomes to result in the 2*n* = 10 female, 11 male karyotypes of the swamp wallaby (*W. bicolor*).

Macropods have experienced extensive genomic reshuffling since they diverged 23 million years ago from a common ancestor with a 2*n* = 22 karyotype [19]. This macropodid ancestral karyotype can be derived from that of the ancestral marsupial by five fissions followed by one fusion and two centromere repositioning events (Figure 3a) [19,72,73,74]. The chromosomal segments involved in these rearrangements overlap with those demonstrated to be highly rearranged amongst the broader marsupial phylogeny [62]. The extent of rearrangement experienced during the evolution of this family has led to macropods being the most comprehensively studied marsupial family at the chromosome level [75]. Diploid numbers vary among macropods from the most derived and lowest diploid number karyotype for a marsupial, consisting of 2*n* = 10 F /11 for the swamp wallaby (*Wallabia bicolor*) to 2*n* = 24 for the banded hare wallaby (*Lagostrophus fasciatus*) [74]. Techniques such as G-banding [72,76,77,78,79,80,81,82], chromosome painting [39,83,84], and FISH with a telomeric probe used to detect chromosome fusions [85,86,87,88,89] have all helped to decipher the genomic reshuffling events among macropods, including a large number of interchromosomal rearrangements. For example, using probes for each tammar wallaby chromosome and FISH with a telomeric probe demonstrated that swamp wallaby chromosome 1 was formed by the fusion of ancestral macropod (AnMac) chromosomes 6, 10, and 4 and an inversion; chromosomes 2 and 3 were the result of a Robertsonian fusion [39,86]. The long arm of the X and Y_2_ is the result of fusion of AnMac chromosomes 2 and 7, with the addition of the AnMac X chromosome making up the short arm of the swamp wallaby X (Figure 3b). 

Unlike dasyurids, rearrangements of macropod chromosomes have often involved the centromeres through either centromere repositioning, Robertsonian fusions, or pericentric inversions [55,89]. Furthermore, several breakpoints reused during macropodid evolution occur at centromeres and have involved five of the same chromosome segments most often rearranged more broadly among marsupials [90]. Interestingly, there is a correlation among centromere satellite sequences, breakpoint reuse, and karyotype convergence amongst nine macropod species, with contractions and expansions of predominant satellites appearing to occur with particular chromosome rearrangements [90]. For instance, in the antilopine wallaroo (*Osphranter antilopinus*) and common wallaroo (*Osphranter robustus*), satellite sat1 was restricted to centromeres separating rearranged chromosomal segments [90]. The centromere, therefore, in macropods appears to contribute in some way to chromosome rearrangement in this family. The study of macropod species hybrids supports this idea as chromosome aberrations in these hybrids are generally associated with the centromere [91,92].

The ability to cross different macropodid species to generate interspecific hybrids makes this family an excellent model group for studying the role of chromosome rearrangements in speciation [93]. The 42 recorded macropod hybrids generated from crosses of species with diploid numbers ranging from 2*n* = 11 to 2*n* = 22 have either been discovered in the wild or bred in captivity [93,94]. The extent to which the fertility of these hybrids is affected varies from complete sterility (more commonly observed in males) to partial fertility in both sexes [93,94]. Macropod hybrids provide an excellent opportunity to study the role of chromosome rearrangements in meiosis and the role, if any, they play in reproductive isolation [93]. 

The centromeres of these hybrids are hotspots of genome instability [91]. Examination of some of the hybrids has revealed destabilization of the centromeres, resulting in chromosome rearrangements such as fissions, isochromosome formation, whole-arm reciprocal translocations, and the formation of minichromosomes [92], as well as the amplification of repetitive sequences associated with macropod centromeres [91,92]. Amplification of the kangaroo endogenous retrovirus (KERV) in a tammar wallaby (2*n* = 16) × swamp wallaby (2*n* = 11) cross was also associated with demethylation of these transposable elements [91]. The propensity of particular genomic regions in these hybrids to be involved in rearrangements could lead to independent derivation of the same rearrangements in different hybrid individuals [95]. If this were to occur in the wild, the chances of homozygous offspring for rearrangements could be feasible within one or a small number of generations [95]. 

### Rock-Wallabies as a Model Group for Studying Speciation

**Figure 4:**
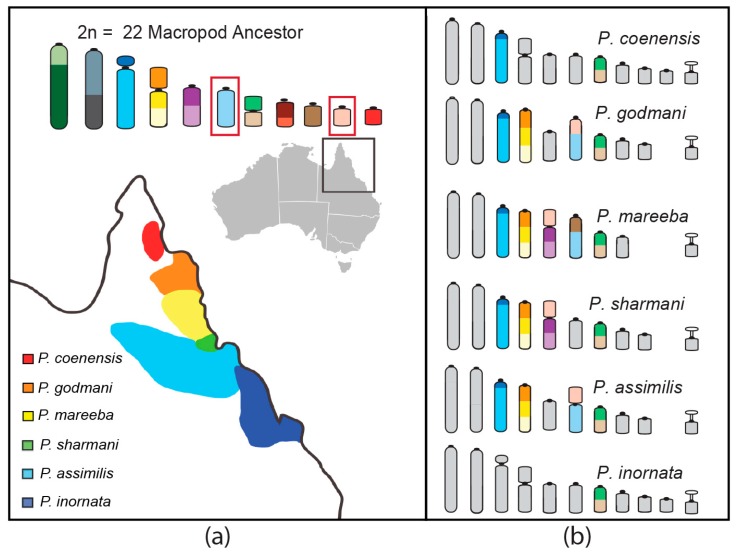
Karyotypic variation amongst six parapatric *Petrogale* species in Queensland. (**a**) The geographic distribution of the six species (adapted from [101]). (**b**) The differences from the ancestral macropod karyotype are highlighted to demonstrate the frequent involvement of ancestral chromosomes 6 and 10 in rearrangements among these species.

The genus *Petrogale* (rock-wallabies) has been put forward as an excellent model system for studying chromosomal speciation because of the extensive genomic reshuffling they have experienced and their habitat specialization to rock outcrops resulting in small and isolated populations [1]. *Petrogale* is the most karyotypically diverse genus of marsupials, consisting of 17 species with 23 chromosomal races [96]. Four species have retained the 2*n* = 22 ancestral macropod karyotype (*P. lateralis*, *P. persephone*, *P. rothschildi*, and *P. xanthopus*) [97]. Rearrangements in other species range from simple to complex but the most interesting group from a speciation perspective is the penicillata group in Queensland (Figure 4). This group of parapatric species (*P. coenensis*, *P. godmani*, *P. mareeba*, *P. sharmani*, *P. assimilis*, *P. inornata*) diverged between 0.5 and 2.7 million years ago [98] and in some respects it would seem that speciation is still in the process of occurring in this genus, which provides an opportunity to potentially distinguish causes from consequences when it comes to the role of chromosome rearrangements in speciation. 

Breeding experiments provided evidence of reproductive isolation occurring between the species within the pencillata group, with male hybrids being infertile and either reduced fertility or infertile female hybrids [94,99]. The extent of chromosomal heterozygosity in these hybrids was related to the degree of issues experienced during spermatogenesis and sex chromosome-autosome associations [100]. Despite these meiotic issues, there may be gene flow between these species as there is extensive allele sharing between some of the most karyotypic divergent pencillata group species [101]. Hence, the penicillata group provides the opportunity to comprehensively assess the contribution of genome sequence and function, as well as chromatin conformation to genomic reshuffling and the role this reshuffling may play in speciation [96].

A combined cytogenetics, genomics, and epigenomics approach is required to test models of speciation and genome evolution in the *Petrogale* system. Current efforts are focused on producing molecular cytogenetic maps for four of the six species to anchor whole-genome sequence assemblies and provide the necessary resources for a thorough analysis of the features of rearranged versus non-rearranged regions and breakpoints. Data suggests that—just as broader marsupial comparisons have shown certain regions to be susceptible to rearrangement—there will be hotspots for breakage in the *Petrogale* genome. Experiments on *Petrogale* cells in culture treated with gamma radiation revealed hotspots for breakage on mitotic chromosomes equivalent to AnMac chromosomes 5 and 6 of *P. mareeba* and *P. assimilis* [89]. Treatment of *P. penicillata* cells with mitomycin C induced centric fusions more frequently with the equivalent of AnMac 10 than any other chromosome [89]. These chromosomes correspond to those commonly involved in rearrangements across marsupials. The integrative breakage model [4] would predict these regions to have an open chromatin conformation so assessing the epigenetic landscape and transcription output of *Petrogale* chromosomes will be a crucial step towards testing this model and determining the features that may be making AnMac chromosomes 5, 6, and 10 susceptible to rearrangement. 

## 5. Conclusions

Marsupials, with their distinctively large chromosomes, provide some excellent opportunities to test models of genome evolution and speciation but to do this there needs to be a closer union between cytogenetics, genomics, and epigenomics. Genomes anchored to chromosomes are the essential resources required to delve deeper into the mechanisms driving genome reshuffling and speciation. It will be particularly interesting to compare and contrast the two families (the dasyurids and macropods) with opposing rates of karyotypic evolution. Until we have anchored genome assemblies, many questions regarding these opposing rates of genomic reshuffling will remain unanswered. With the proper genomic resources and with comparisons between these two families, it may be possible to determine whether chromosome rearrangements are a cause or consequence of speciation.
